# Genomic Characterization of *Salmonella enterica* Isolates From Retail Meat in Beijing, China

**DOI:** 10.3389/fmicb.2021.636332

**Published:** 2021-04-07

**Authors:** Na Lyu, Yuqing Feng, Yuanlong Pan, Hua Huang, Yan Liu, Chenyu Xue, Baoli Zhu, Yongfei Hu

**Affiliations:** ^1^CAS Key Laboratory of Pathogenic Microbiology and Immunology, Institute of Microbiology, Chinese Academy of Sciences, Beijing, China; ^2^State Key Laboratory of Animal Nutrition, College of Animal Science and Technology, China Agricultural University, Beijing, China; ^3^Beijing Products Quality Supervision and Inspection Institute, Beijing, China; ^4^Beijing Municipal Center for Food Safety Monitoring and Risk Assessment, Beijing, China

**Keywords:** *Salmonella enterica*, retail meat, next-generation sequencing, serotype, genome plasticity, virulence gene, antimicrobial resistance gene, MLST

## Abstract

*Salmonella enterica* remains one of the leading causes of foodborne bacterial disease. Retail meat is a major source of human salmonellosis. However, comparative genomic analyses of *S. enterica* isolates from retail meat from different sources in China are lacking. A total of 341 *S. enterica* strains were isolated from retail meat in sixteen districts of Beijing, China, at three different time points (January 1st, May 1st, and October 1st) in 2017. Comparative genomics was performed to investigate the genetic diversity, virulence and antimicrobial resistance gene (ARG) profiles of these isolates. The most common serotype was *S.* Enteritidis (203/341, 59.5%), which dominated among isolates from three different time points during the year. Laboratory retesting confirmed the accuracy of the serotyping results predicted by the *Salmonella In Silico* Typing Resource (SISTR) (96.5%). The pangenome of the 341 *S. enterica* isolates contained 13,931 genes, and the core genome contained 3,635 genes. Higher *Salmonella phage 118970 sal3* (219/341, 64.2%) and *Gifsy-2* (206/341, 60.4%) prevalence contributed to the diversity of the accessory genes, especially those with unknown functions. IncFII(S), IncX1, and IncFIB(S) plasmid replicons were more common in these isolates and were major sources of horizontally acquired foreign genes. The virulence gene profile showed fewer virulence genes associated with type III secretion systems in certain isolates from chicken. A total of 88 different ARGs were found in the 341 isolates. Three beta-lactamases, namely, *bla*_*CTX*__–__*M*__–__55_ (*n* = 15), *bla*_*CTX*__–__*M*__–__14_ (*n* = 11), *and bla*_*CTX*__–__*M*__–__65_ (*n* = 11), were more prevalent in retail meats. The emergence of *qnrE1* and *bla*_*CTX*__–__*M*__–__123_ indicated a potential increase in the prevalence of retail meats. After the prohibition of colistin in China, three and four isolates were positive for the colistin resistance genes *mcr-1.1* and *mcr-9*, respectively. Thus, we explored the evolution and genomic features of *S. enterica* isolates from retail meats in Beijing, China. The diverse ARGs of these isolates compromise food security and are a clinical threat.

## Introduction

Foodborne diseases caused by *Salmonella* are an important public health concern. More than 2600 *Salmonella* serovars have been classified (Guibourdenche et al., 2010). Human-adapted typhoid serovars (commonly *Salmonella enterica* serovars Typhi and Paratyphi A) cause the severe systemic disease typhoid fever, while most human non-typhoidal *Salmonella* (NTS) infections are attributed to broad-host-range serotypes that cause self-limiting gastroenteritis (Coburn et al., 2007).

China has a high incidence of salmonellosis, and retail meat or meat products are recognized as the main reservoirs of human foodborne NTS (Ma et al., 2017; Zhang Z. et al., 2018; Li et al., 2019). Accordingly, high rates of *Salmonella* contamination in retail meats such as pork, beef, mutton, and duck meat have been reported in several places in China. A high prevalence of *Salmonella* contamination has been observed in chicken (63.6%), pork (73.1%), and duck meat (41.4%) in Guangdong, China (Zhang L. et al., 2018; Chen et al., 2020). In a study in which a total of 807 retail meat samples were collected from most provincial capitals in China between July 2011 and June 2016, 19.7% of the samples tested positive for *Salmonella*. The highest contamination rate occurred in pork (37.3%), followed by beef (16.1%) and mutton (10.9%) (Yang et al., 2019).

The pathogenesis of *Salmonella* is associated with the expression of virulence-associated determinants in *Salmonella* pathogenicity islands (SPIs) (Groisman and Ochman, 1996). To date, 24 different SPIs have been identified, but not all of them have been experimentally validated (Lerminiaux et al., 2020). Two distinct type III secretion systems (TTSSs) encoded by SPI-1 and SPI-2 are considered central to the pathogenicity of NTS (Suez et al., 2013). SPI-1 genes are responsible for the invasion of host cells and for the regulation of the host immune response, while SPI-2 contributes to intracellular survival and replication (El Ghany et al., 2016; Lou et al., 2019).

In severe cases of salmonellosis, antibiotics are typically prescribed for treatment; however, the emergence of multidrug-resistant (MDR) *S. enterica* strains has raised global concern, due to an increase in the mortality rate of infected patients (Gong et al., 2019; Zhan et al., 2019). In particular, extended-spectrum beta-lactamase-producing *Salmonella* strains have been frequently isolated from food animals in many countries (Zhao et al., 2017). High rates of antimicrobial resistance to sulfisozazole (76.1%), tetracycline (75.3%), ampicillin (48.0%), and ofloxacin (44.7%) have been found in *Salmonella* isolates from retail chicken and pork meat samples in Guangdong, China (Zhang L. et al., 2018). In another study, among 218 *Salmonella* isolates obtained from retail meat and meat products in China, 181 (83.0%) were resistant to at least one class of antimicrobials, and 128 (58.7%) were resistant to at least three classes (Yang et al., 2019).

Next-generation sequencing technology provides an unparalleled, powerful tool for the characterization of infectious agents such as *Salmonella* and the investigation of foodborne outbreaks (Mather et al., 2013; den Bakker et al., 2014). Genomic analysis can provide improved and exhaustive data related to pathogen genotypic characteristics and can allow the identification of virulence determinants, antimicrobial resistance genes and serotypes. The classification of *Salmonella* based on genome-sequencing technologies is especially important for epidemiological investigations and has been implemented at the PulseNet (Nadon et al., 2017).

Beijing is the second largest city in China, with a population of over 20 million people. It has been estimated that the total annual meat consumption in Beijing is more than 1.3 million tons (Shi et al., 2015). However, there is limited available information on the surveillance and large-scale genomics studies of *Salmonella* in meats from retail markets in Beijing. This study is, to the best of our knowledge, the first comparative genomic study of *S. enterica* isolates from different sources in Beijing.

## Materials and Methods

### Experimental Design and Sample Collection

To explore the genomic characteristics of *S. enterica* isolates from retail meat in Beijing, China, we collected 1,234 raw samples of chicken meat (*n* = 688), duck meat (*n* = 108), pork (*n* = 220), beef (*n* = 163) and mutton (*n* = 55) from retail markets located in all sixteen districts of Beijing, China. To study the prevalence of *Salmonella* over a year in meats, these samples were collected at three different time points (January 1st, May 1st, and October 1st) in 2017.

### *Salmonella* Isolation

The isolation and identification of *Salmonella* were performed according to procedures described in the National Standards of the People’s Republic of China (GB 4789.1-2016). Briefly, a 25-g raw meat sample was placed in 225 mL of buffered peptone water (Land Bridge Technology Co., Ltd., Beijing, China) and incubated at 37°C for 8 h. One-milliliter aliquots of the pre-enriched cultures were transferred to 10 mL of tetrathionate broth (TTB, Land Bridge Technology Co., Ltd., Beijing, China) or selenite cystine broth (SCB, Land Bridge Technology Co., Ltd., Beijing, China). After incubation at 42°C for 18 h in TTB and SCB, one loopful of each broth was streaked onto bismuth sulfite (BS, Land Bridge Technology Co., Ltd., Beijing, China) or xylose lysine deoxycholate agar (XLD, Land Bridge Technology Co., Ltd., Beijing, China). The BS plates were incubated at 37°C for 40–48 h, and the XLD plates were incubated at 37°C for 18–24 h. After incubation, the plates were examined for morphologically typical *Salmonella* colonies. Among the positive samples, one presumptive *Salmonella* isolate was picked and confirmed by sequencing the 16S rRNA gene (Frank et al., 2008).

### Serotyping and Antimicrobial Susceptibility Testing

*Salmonella* isolates were serotyped via the slide agglutination assay according to the instructions provided by the manufacturer of the *Salmonella* antisera (Statens Serum Institute, Copenhagen, Denmark). The serovars of the isolates were determined using the Kauffmann-White scheme (Grimont and Weill, 2007).

Antimicrobial susceptibility testing of *Salmonella* isolates was performed using the agar dilution method for ampicillin, ampicillin-sulbactam, piperacillin, piperacillin-tazobactam, cefuroxime, cefotetan, ceftazidime, ceftriaxone, cefepime, aztreonam, imipenem, amikacin, gentamicin, and tobramycin (Sigma, St. Louis, MO, United States). *Escherichia coli* ATCC 25922 was used as a quality control strain in each run. The breakpoints for determining susceptibility or resistance to antimicrobials were determined by the guidelines of the Clinical Laboratory Standard Institute (CLSI, 2018).

### DNA Sequencing, Assembly, and Annotation

For whole-genome sequencing (WGS), the genomic DNA of the isolates was extracted by using the QIAamp DNA Mini Kit (Qiagen, Hilden, Germany). A DNA library was constructed using a NEXTflex^®^ rapid DNA-Seq Kit according to the manufacturer’s instructions (Illumina, San Diego, United States). In brief, one paired-end library with an insert size of 350 bp for each sample (Tan et al., 2019) was constructed and sequenced with a 150-bp read length from each end on the Illumina HiSeq 2500 platform (Illumina, San Diego, United States). Adaptors were clipped from the Illumina HiSeq-generated sequencing reads with Trimmomatic (Bolger Anthony et al., 2014). The reads were assembled using Unicycler (v0.4.7) with the default parameters (Wick et al., 2017), including short-read assembly via SPAdes (v3.13.0) (Anton et al., 2012) and polishing via Pilon (Walker et al., 2014). The genomes were annotated using Prokka (v1.13.3) (Seemann, 2014), which uses Prodigal (v2.6.3) (Hyatt et al., 2010) to identify open reading frames.

### *In silico* Analysis of *Salmonella* Genomes

Core genome and pangenome prediction was carried out using Roary software (v3.11.2) (Page et al., 2015), and the core genome was defined by including both hard-core and soft-core genes (i.e., genes present in more than 95.0% of isolates) (Livingstone et al., 2018). The core gene and accessory gene functional annotations of *Salmonella* were carried out using the eggNOG platform (v4.5) (Huerta-Cepas et al., 2016). To determine clonality or genetic relatedness between the isolates, phylogenetic reconstruction was calculated using kSNP3 (v3.0) with a kmer size of 19 (Gardner et al., 2015). A maximum likelihood (ML) tree was calculated from 277,664 core SNPs using kSNP3. SNP distance based on the 277,664 core SNPs was calculated using the snp-dists^1^. In addition to serotyping, *in silico* analysis by MLST was also performed using the *Salmonella In Silico* Typing Resource (SISTR) on the basis of whole-genome assemblies (Yoshida et al., 2016). We identified putative plasmid sequences with the Basic Local Alignment Search Tool (BLAST, v2.9.0+) by searching PlasmidFinder database replicons against our dataset with BLASTn (Altschul et al., 1990). A sequence identity of 80% was used as a cutoff for calling a specific replicon as present or absent. Prophage predictions were carried out using PHASTER to explore prophages (Arndt et al., 2016). Virulence genes were identified by BLAST searches against the latest version of the Virulence Factor Database (VFDB) (Chen et al., 2016) with cutoff values of ≥ 80% identity and ≥ 50% coverage. The partial plasmid sequences from the draft genomes were predicted using MOB-suite (Robertson and Nash, 2018) and annotated against the VFDB with the same criteria. ARG analysis was performed by using ResFinder v4.0 (Bortolaia et al., 2020). A sequence identity of 80% and ≥ 50% coverage were used as the cutoffs for calling an ARG as present or absent. Mobile genetic elements (MGEs) in *mcr*-positive isolates were further investigated according to the results of MOB-suite.

### Statistical Analysis and Visualization

The Wilcoxon rank-sum test was used to identify differences in the number of ARGs and VFs. The Kruskal-Wallis test was performed when comparing samples from more than two groups. The statistical significance of the categorical variables was assessed using the chi-square test. The phylogenetic tree was edited and exported using the iTOL tool (Letunic and Bork, 2019). All statistical analyses were performed using R software (v3.6.3). The R package ggplot2 was used for data visualization in figures in the present study (Valero-Mora, 2010).

## Results

### Isolation of *Salmonella* and Sequence Statistics

**FIGURE 1:**
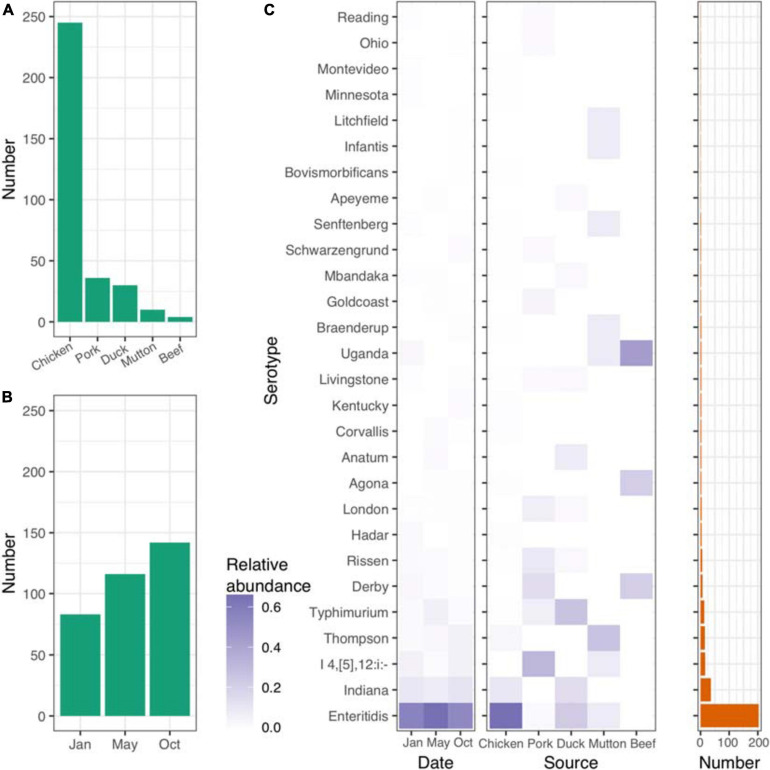
Distribution of isolates and *Salmonella* serovars. **(A)** Number of isolates from five different retail meats. **(B)** Number of isolates from different sampling time points. **(C)** Distribution of serovars across different time points and retail meats. Left panel, time points; middle panel, retail meats; right panel, number of each serovar.

In the present study, we isolated 341 *Salmonella* strains from retail meat samples, including chicken, pork, duck, beef and mutton specimens, collected from sixteen districts of Beijing, China, at three different time points (January 1st, May 1st, and October 1st) in 2017 (Figures 1A,B and Supplementary Table 1). The raw whole-genome sequence data totaled more than ∼800 Mbp for each isolate (Supplementary Table 1). The average lengths of the genomes ranged from 4.50 to 5.15 Mbp, with an overall average length of 4.78 Mbp (Supplementary Table 1).

### Prevalence of Serotypes and Sequence Types (STs) Associated With Different Sources and Times

*Salmonella* serotyping remains the gold-standard tool for the classification of *Salmonella* isolates. To verify the accuracy of the results from the *in silico* analysis, we randomly selected 85 isolates for serotyping. SISTR software showed a consistency of 96.5% (82/85). Among the three isolates that were poorly predicted by SISTR, two *S.* Typhimurium isolates were predicted to belong to the serovar *S.* I 4,[5],12:i- (Supplementary Table 2). Based on the serovar prediction results from SISTR (Supplementary Table 3), we further explored the connections between the serovars and the corresponding epidemiological information. In total 28 different serovars were identified for the 341 isolates recovered. Most of the isolates were of chicken origin and belonged to *S.* Enteritidis (Figure 1C and Supplementary Table 4). The frequency of *S.* Enteritidis was stable even at different time points throughout 2017 (Figure 1C and Supplementary Table 5). However, different sources shared different patterns of serovars. *S.* Enteritidis, *S.* I 4,[5],12: i-, *S.* Typhimurium, *S.* Thompson and *S.* Uganda were the dominant serovars in chicken, pork, duck, mutton, and beef, respectively (Supplementary Table 6).

We further explored the STs of the isolates using SISTR. A total of 33 different STs were identified among the 341 isolates recovered. The most frequent ST was ST11 (203/341, 59.5%, Supplementary Table 7), which also belonged to the *S.* Enteritidis serovar. Some STs, such as ST17 (*n* = 36), ST34 (*n* = 17), ST26 (*n* = 15), ST19 (*n* = 9), ST40 (*n* = 7), and ST469 (*n* = 6), were also common in the isolates. Twenty-four STs (ratio < 1.0%) were rare in the isolates from retail meat (Supplementary Table 8).

### Phylogenetic Analysis Revealed Multiple Origins of *Salmonella*

**FIGURE 2:**
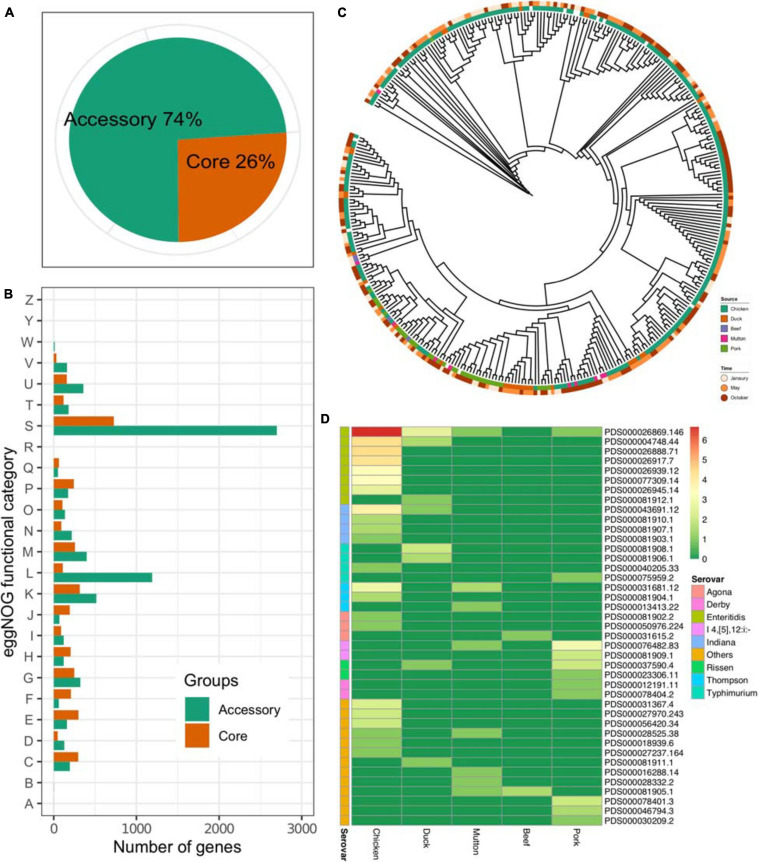
Core genome of *Salmonella*. **(A)** Proportions of accessory genes and core genes in the pangenome. **(B)** Core and accessory genome functional annotation. RNA processing and modification, A; chromatin structure and dynamics, B; energy production and conversion, C; cell cycle control, cell division, chromosome partitioning, D; amino acid transport and metabolism, E; nucleotide transport and metabolism, F; carbohydrate transport and metabolism, G; coenzyme transport and metabolism, H; lipid transport and metabolism, I; translation, ribosomal structure and biogenesis, J; transcription, K; replication, recombination and repair, L; cell wall/membrane/envelope biogenesis, M; cell motility, N; posttranslational modification, protein turnover, chaperones, O; inorganic ion transport and metabolism, P; secondary metabolites biosynthesis, transport and catabolism, Q; general function prediction only, R; function unknown, S; signal transduction mechanisms, T; intracellular trafficking, secretion, and vesicular transport, U; defense mechanisms, V; extracellular structures, W; nuclear structure, Y; cytoskeleton, Z. **(C)** An ML tree constructed based on the core SNPs (*n* = 277,664). **(D)** Prevalence of the SNP clusters from different sources. Color: the number of the isolates (log2-transformed).

The results showed that the pangenome of the 341 isolates contained a total of 13,931 genes, while the core genome (3,625 genes) accounted for approximately one-quarter of the pangenome (Figure 2A). On the other hand, most of the *Salmonella* genome was conserved, considering the total number of genes in each isolate (Supplementary Table 1). The accessory genome harbored more genes associated with the “[S]Function unknown” (Chi-square test, *p* < 2.2 × 10^16^), “[L]Replication, recombination and repair” (Chi-square test, *p* < 2.2 × 10^16^, *p* < 2.2 × 10^16^), and “[K]Transcription” (Chi-square test, *p* = 0.027) categories (Figure 2B). An ML tree was generated on the basis of the core SNPs (Figure 2C). Considering the prevalence of the serovar from different sources, isolates of the same serovar recovered from different sources were further analyzed on four serovars, i.e., *S.* Enteritidis, *S.* Indiana, *S.* Thompson, and *S.* Typhimurium. The results showed that the isolates of *S.* Typhimurium from duck and pork showed longer inter-SNP distance than the intra-SNP distance (*p* = 7.57 × 10^9^), while the others were not (Supplementary Figure 1). According to the data from the NCBI, 291 out of 341 isolates could be assigned to known SNP clusters (*n* = 41). Compared with pork (*n* = 11), duck (*n* = 8), mutton (*n* = 8), and beef (*n* = 2), the number of SNP clusters in chickens was higher (*n* = 22) (Figure 2D). In addition, there were eight SNP clusters with isolates from multiple sources, among which two SNP clusters (PDS000026869.146 and PDS000004748.44) belonged to *S.* Enteritidis. Notably, the most common SNP cluster, PDS000026869.146, contained 107 isolates having different sources; 100 were from chicken, 5 from duck, 1 from mutton, and 1 from pork. Additionally, there were two SNP clusters, PDS000004748.44 and PDS000043691.12, contained strains from both chicken and duck. These results strongly suggested the common source of S*almonella* contamination in retail meat.

### Isolates From Retail Meat Presented Different MGEs

**FIGURE 3:**
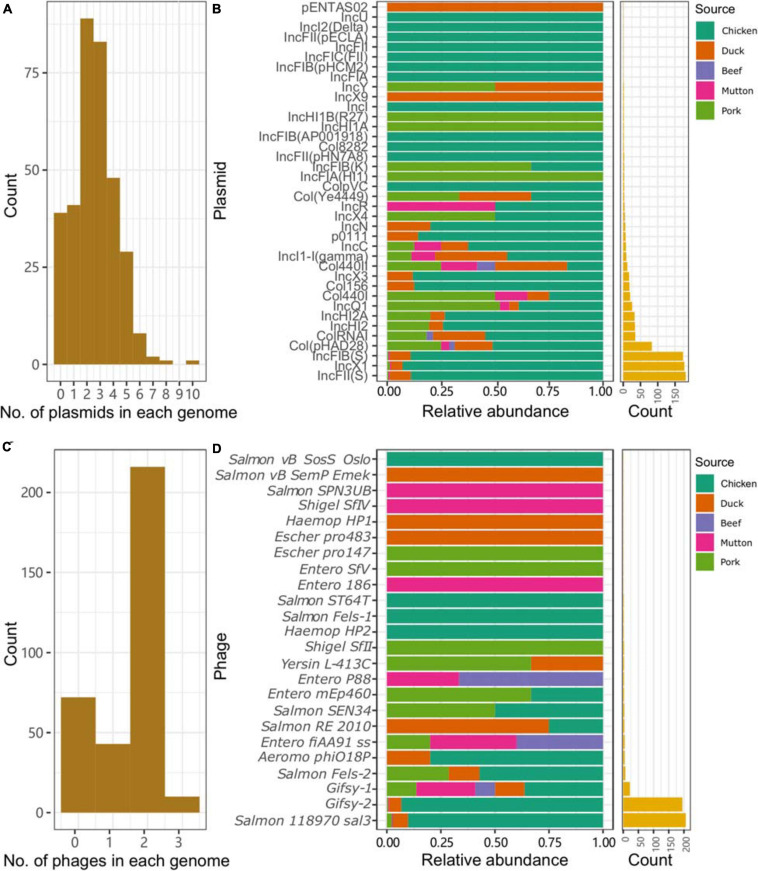
Plasmids and prophages in the *Salmonella* genome. **(A)** Number of plasmids in each genome. **(B)** Distribution of different plasmids in the genomes of *Salmonella* isolates from different retail meats. **(C)** Number of prophages in each genome. **(D)** Distribution of different prophages in the genomes of *Salmonella* isolates from different retail meats.

Plasmid replicons were widely present among the isolates. Only 11.4% (39/341) of the isolates did not harbor any plasmids in their genomes. A total of 885 plasmid replicons were found among the 341 isolates, with a maximum of ten plasmid replicons observed in a single isolate (Figure 3A). Plasmid replicons of IncFII(S), IncX1, and IncFIB(S) were the three most common types of replicons (*n* = 181, 177, and 173, respectively; Figure 3B and Supplementary Table 9). The less common types, such as Col(pHAD28), ColRNAI, IncHI2, IncHI2A, IncQ1, and Col4401, were more likely to be present in the isolates from pork upon considering the total number of isolates from chicken (*n* = 245) and pork (*n* = 36). Isolates from retail meat showed differences in the prevalence of plasmid replicons at different time points (Supplementary Figure 2A). Specifically, strains isolated in January and May showed a higher prevalence of IncX1. Strains isolated in May and October showed a higher prevalence of IncFII(S) and IncFIB(S). For different serovars, *S.* Indiana harbored the largest number of plasmid types (*n* = 21), compared with the other serovars (Supplementary Figure 3). At the same time, isolates from *S.* Enteritidis were more prone to carry the plasmid replicons of IncFIB(S), IncFII(S) and IncX1.

Prophages were also highly prevalent among the isolates. Only 72 isolates did not contain any intact prophages in their genomes. A total of 505 prophages were found among the 341 isolates, with a maximum of three observed in a single isolate (Figure 3C). More than half of the isolates (216/341, 63.3%) harbored two intact prophages. Interestingly, 203 isolates harbored both *Gifsy-2* and *Salmon 118970 sal3* (Figure 3D). The other 22 kinds of prophages were rare in the isolates from retail meat (Supplementary Table 10). The third most common prophage, *Gifsy-1*, was more likely to be present in isolates from hosts other than chicken. Strains isolated in May and October showed a higher prevalence of *Salmon 118970 sal3* and *Gifsy-2* prophages (Supplementary Figure 2B). For different serovars, *S.* I 4,[5],12:i:- harbored the largest number of prophage types (*n* = 10), compared with the other serovars (Supplementary Figure 4). At the same time, isolates from *S.* Enteritidis were more prone to contain *Gifsy-2* and *Salmon 118970 sal3*. In addition, the prophages in the isolates were also found to be able carry ARGs (*n* = 31) and VFs (*n* = 335) (Supplementary Tables 11, 12).

### Isolates From Chicken Showed Fewer Virulence Factors (VFs)

**FIGURE 4:**
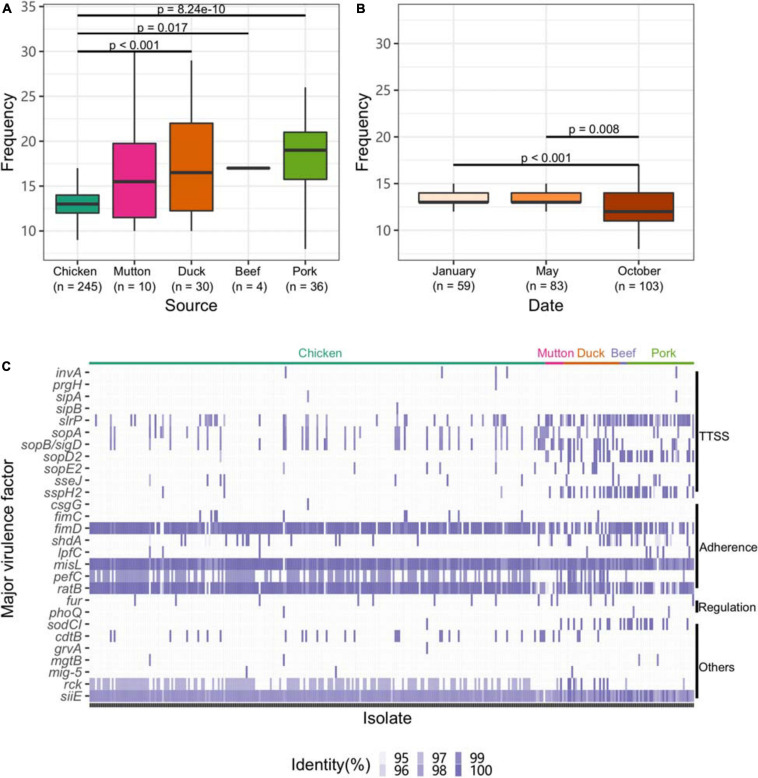
Profile of VFs in the *Salmonella* genome. **(A)** Numbers of VFs in the genomes of *Salmonella* isolates from different retail meats. **(B)** Numbers of VFs in the genomes of *Salmonella* isolates from different sampling time points. **(C)** Major VF profile in the *Salmonella* genome. TTSS, Type III secretion system.

To explore the virulence gene profile of *Salmonella*, we subjected the genomes to BLAST searches against the full VFDB dataset (Supplementary Table 13). Compared with other sources, isolates from chicken harbored fewer VFs (*p* < 0.05, Figure 4A). Strains isolated from chicken in October exhibited a lower frequency of VFs than those isolated from chicken at the other two time points (*p* < 0.05, Figure 4B).

Eleven genes associated with the TTSS were found among the isolates. Among them, *slrP*, *sopA*, and *sopB*/*SopD* were common in the isolates. Genes associated with adherence (*fimD*, *misL*, and *ratB*) and *siiE* were ubiquitously distributed among the isolates. Isolates from chicken harbored fewer genes associated with the TTSS than those from other hosts (Figure 4C). Additionally, the strains isolated from chicken harbored more genes associated with adherence, such as *fimD* and *pefC*. We further analyzed the VF categorization based on chromosomes and plasmids. One VF, *rck*, may be carried by plasmids belonging to the plasmid replicons of IncFII(S), IncX1 and IncFIB(S) (Supplementary Figure 5). Unlike the plasmid and the prophage, the isolates from different serovars almost shared the same patterns of major VFs (Supplementary Figure 6).

### ARG Profiles of *Salmonella*

Antimicrobial susceptibility testing was performed in 117 isolates (Supplementary Table 14). The isolates showed a higher rate of resistance to ampicillin (67.5%, Table 1). All 117 isolates were susceptible to cefotetan, imipenem and piperacillin-tazobactam.

**TABLE 1:** Antimicrobial resistance of 117 *Salmonella* isolates recovered from retail meat.

Antimicrobial^a^	Resistance breakpoint^b^	No. of resistant isolates (%)
AMP	32	79(67.5%)
SAM	32/16	77(65.8%)
PIP	128	68(58.1%)
CXM	32	34(29.1%)
CRO	4	24(20.5%)
ATM	16	20(17.1%)
PEP	16	16(13.7%)
CAZ	16	15(12.8%)
CTT	64	0(0.0%)
IPM	4	0(0.0%)
TZP	128/4	0(0.0%)
GEN	16	24(20.5%)
TOB	16	22(18.8%)
AMK	64	10(8.5%)

*^a^Antimicrobial abbreviations: ampicillin (AMP), ampicillin-sulbactam (SAM), piperacillin (PIP), cefuroxime (CXM), ceftriaxone (CRO), aztreonam (ATM), cefepime (PEP), ceftazidime (CAZ), cefotetan (CTT), imipenem (IPM), piperacillin-tazobactam (TZP), gentamicin (GEN), tobramycin (TOB), amikacin (AMK).*

*^b^Breakpoints followed the instructions of the Clinical and Laboratory Standards Institute.*

**FIGURE 5:**
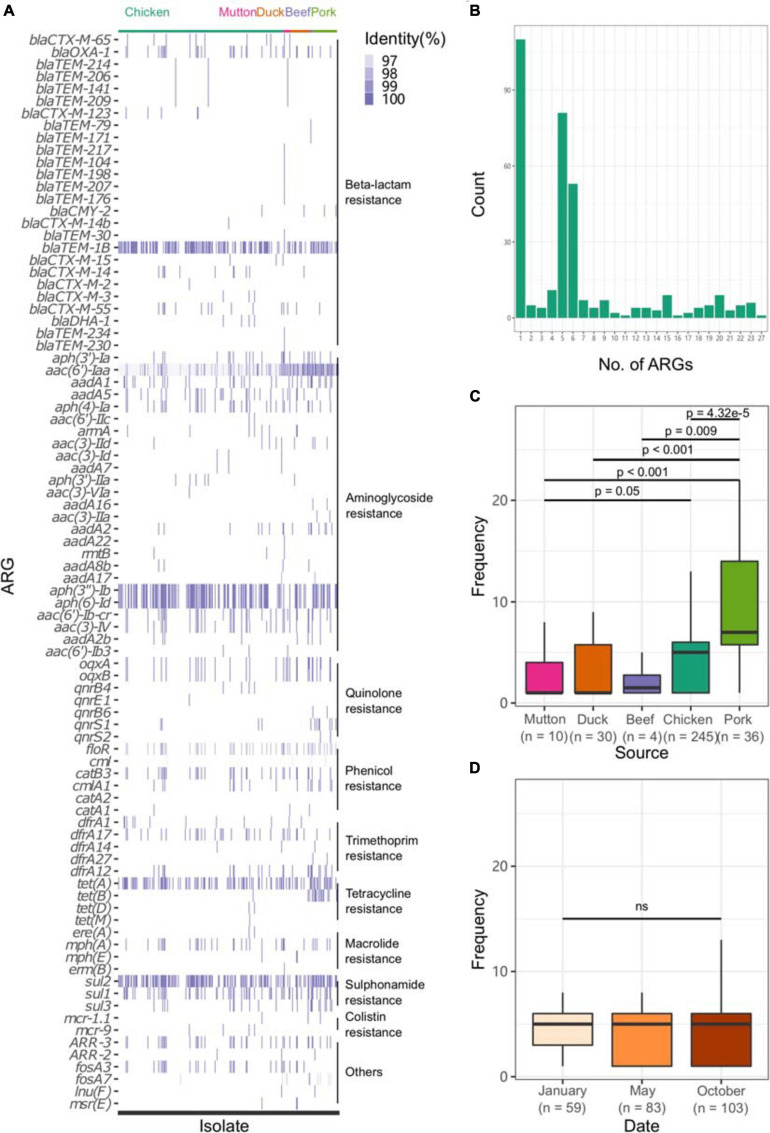
Profile of ARGs in the *Salmonella* genome. **(A)** ARG profile in the *Salmonella* genome. **(B)** Number of ARGs in each genome. **(C)** Number of ARGs in the genomes of *Salmonella* isolates from different retail meats. **(D)** Number of ARGs in the genomes of *Salmonella* isolates from different sampling time points.

All sequences of the 341 genomes were screened against the ResFinder database for the presence of resistance genes. A total of 88 different ARGs were found in the 341 isolates (Figure 5A). Most of the ARGs belonged to the categories of beta-lactam resistance (*n* = 26) and aminoglycoside resistance (*n* = 25). Among the CTX-M-type beta-lactamases, eight *bla*_*CTX*__–__*M*_ genes (*bla*_*CTX*__–__*M*__–__2_, *bla*_*CTX*__–__*M*__–__3_, *bla*_*CTX*__–__*M*__–__14_, *bla*_*CTX*__–__*M*__–__1__4b_, *bla*_*CTX*__–__*M*__–__15_, *bla*_*CTX*__–__*M*__–__55_, *bla*_*CTX*__–__*M*__–__65_, *bla*_*CTX*__–__*M*__–__123_) were identified. The most prevalent genes detected were *bla*_*CTX*__–__*M*__–__55_ (*n* = 15), *bla*_*CTX*__–__*M*__–__14_ (*n* = 11), and *bla*_*CTX*__–__*M*__–__65_ (*n* = 11). Notably, all the isolates were positive for the aminoglycoside resistance gene *aac(6′)-Iaa*, and half of the isolates possessed a sulfonamide resistance gene (*sul2*), a beta-lactam resistance gene (*bla_*TEM*–1B_*) and aminoglycoside resistance genes [*aph(6)-Id* and *aph(3″)-Ib*] (Supplementary Table 15). Seven kinds of plasmid-mediated quinolone resistance (PMQR) genes were found among the isolates, namely, *oqxA* (*n* = 31), *oqxB* (*n* = 29), *qnrB4* (*n* = 4), *qnrB6* (*n* = 2), *qnrE1* (*n* = 1), *qnrS1* (*n* = 12), and *qnrS2* (*n* = 2). In addition, three and four isolates were positive for the colistin resistance genes *mcr-1.1* and *mcr-9*, respectively (Table 1). All *mcr-9*-positive isolates were isolated from chicken. Two of the *mcr-1.1*-positive isolates were isolated from pork, and the third was isolated from chicken. In two isolates, the IncX4 plasmid replicon was predicted, which was genetically linked with the *mcr-1.1* gene on the same contig (Supplementary Table 16). Another *mcr-1.1* gene was associated with the IncI2 plasmid replicon. The *mcr-9* gene was associated with the IncHI2A/rep_cluster_1088 plasmid replicon. Due to the high prevalence of genes associated with aminoglycoside resistance and beta-lactam resistance, all the isolates harbored at least one ARG in their genomes (Figure 5B). One isolate, SLM309, contained 27 ARGs, which may be involved in resistance to antimicrobials from the beta-lactam, aminoglycoside, phenicol, trimethoprim, macrolide, sulfonamide, rifampicin, and fosfomycin categories (Supplementary Table 17). The strains isolated from pork harbored more ARGs than the others (Figure 5C, *p* < 0.05). The number of ARGs in the *Salmonella* genomes from chicken did not change with time (Figure 5D). The isolates in the present study harbored more ARGs and VFs (Supplementary Figure 7) than those of the other four major serovars. The isolates harboring the highest number of ARGs tended to belong to *S.* Indiana, which may be due to the presence of different kinds of plasmids in the isolates (Table 2).

**TABLE 2:** Sources and serovar, ARG, VF, and ST profiles of some *Salmonella* isolates.

Isolate	Source	Serovar	ST	Replicons	Prophage	VFs	ARGs
SLM26	Pork	I 4,[5],12:i:-	34	Col(pHAD28), ColRNAI, IncHI2, IncHI2A, IncX4		*fimD*, *sodCI*, *sspH2*, *slrP*, *siiE*, *misL*, *shdA*, *ratB*, *sopD2*	*ARR-3*, *aac(3)-IV*, *aac(6′)-Iaa*, *aac(6′)-Ib-cr*, *aadA1*, *aadA2b*, *aph(3′)-Ia*, *aph(4)-Ia*, *blaOXA-1*, *catB3*, *cmlA1*, *dfrA12*, *floR*, *mcr-1.1*, *oqxA*, *oqxB*, *sul1*, *sul2*, *sul3*, *tet(B)*
SLM52	Chicken	Indiana	17	Col156, IncHI2, IncHI2A, IncQ1		*fimD*, *siiE*, *cdtB*, *sopA*, *misL*, *ratB*	*aac(3)-IId*, *aac(3)-IV*, *aac(6′)-Iaa*, *aadA1*, *aadA2b*, *aph(3″)-Ib*, *aph(3′)-Ia*, *aph(4)-Ia*, *aph(6)-Id*, *blaCTX-M-65*, *blaTEM-1B*, *catA1*, *cmlA1*, *dfrA12*, *floR*, *fosA3*, *oqxA*, *oqxB*, *rmtB*, *sul1*, *sul2*, *sul3*, *tet(A)*
SLM65	Chicken	Thompson	26	IncHI2, IncHI2A, p0111	Gifsy-1	*siiE*, *fimC*, *misL*, *ratB*, *slrP*, *fimD*	*ARR-3*, *aac(3)-IV*, *aac(6′)-Iaa*, *aac(6′)-Ib-cr*, *aadA1*, *aadA2b*, *aph(3″)-Ib*, *aph(3′)-Ia*, *aph(4)-Ia*, *aph(6)-Id*, *blaCTX-M-14*, *blaCTX-M-55*, *blaOXA-1*, *catB3*, *cmlA1*, *dfrA12*, *floR*, *fosA3*, *mph(A)*, *sul1*, *sul2*, *sul3*, *tet(A)*
SLM68	Chicken	Thompson	26	IncHI2, IncHI2A, p0111	Gifsy-1	*siiE*, *fimC*, *misL*, *ratB*, *slrP*, *fimD*	*ARR-3*, *aac(3)-IV*, *aac(6′)-Iaa*, *aac(6′)-Ib-cr*, *aadA1*, *aadA2b*, *aph(3″)-Ib*, *aph(3′)-Ia*, *aph(4)-Ia*, *aph(6)-Id*, *blaCTX-M-14*, *blaCTX-M-55*, *blaOXA-1*, *catB3*, *cmlA1*, *dfrA12*, *floR*, *fosA3*, *mph(A)*, *sul1*, *sul2*, *sul3*, *tet(A)*
SLM134	Duck	Indiana	17	IncHI2, IncHI2A, IncQ1, p0111		*fimD*, *siiE*, *sopB/sigD*, *cdtB*, *sopA*, *misL*, *ratB*	*ARR-3*, *aac(3)-IV*, *aac(6′)-Iaa*, *aac(6′)-Ib-cr*, *aadA5*, *aph(3″)-Ib*, *aph(3′)-Ia*, *aph(4)-Ia*, *aph(6)-Id*, *blaCTX-M-65*, *blaOXA-1*, *blaTEM-1B*, *catA1*, *catB3*, *dfrA17*, *floR*, *fosA3*, *oqxA*, *oqxB*, *sul1*, *sul2*, *tet(A)*
SLM144	Duck	Indiana	17	–		*fimD*, *siiE*, *sopB/sigD*, *cdtB*, *sopA*, *misL*, *ratB*	*ARR-3*, *aac(3)-IV*, *aac(6′)-Iaa*, *aac(6′)-Ib-cr*, *aadA5*, *aph(3″)-Ib*, *aph(4)-Ia*, *aph(6)-Id*, *armA*, *blaCTX-M-55*, *blaOXA-1*, *catB3*, *dfrA17*, *floR*, *fosA3*, *mph(E)*, *msr(E)*, *oqxA*, *oqxB*, *sul1*, *sul2*, *tet(A)*
SLM145	Duck	Indiana	17	Col156		*fimD*, *siiE*, *cdtB*, *sopA*, *misL*, *ratB*	*ARR-3*, *aac(6′)-Iaa*, *aac(6′)-Ib-cr*, *aadA1*, *aadA2b*, *aadA5*, *aph(3″)-Ib*, *aph(6)-Id*, *armA*, *blaOXA-1*, *catB3*, *cmlA1*, *dfrA17*, *floR*, *fosA3*, *mph(E)*, *msr(E)*, *oqxA*, *oqxB*, *sul1*, *sul2*, *sul3*, *tet(A)*

## Discussion

*Salmonella* remains the leading cause of reported bacterial foodborne disease in China. Retail meats and meat products are recognized as one of the major sources of human salmonellosis (Yang et al., 2019). To investigate the prevalence of *Salmonella* in retail meat available on the market throughout the year, we isolated 341 *Salmonella* strains from Beijing in 2017 and investigated their genomes. To the best of our knowledge, this is the first systematic study reporting the genomic characterization of *Salmonella* isolates from retail meat in China.

Among the various kinds of retail meat, chicken shows the highest rate of *Salmonella* contamination (Zhao et al., 2001; Yang et al., 2010). Most of the 341 isolates examined in the present study (71.8%, 245/341) came from chicken. The limited sample sizes obtained for beef (*n* = 4) and mutton (*n* = 10) made it difficult to draw a clear conclusion about the other kinds of retail meats, which showed a shortage at the time of the present study.

Most of the retail meat samples from which *Salmonella* was isolated were imported from at least eight provinces outside of Beijing in China (data not shown). This may explain why the ML tree and the SNP clusters revealed multiple origins of the isolates. Interestingly, the major SNP cluster found in the present study contained many strains from clinical infections in the following years in China, indicating the need for further study on the spread of strains associated with this SNP cluster. The limited sample size may lead to the high one-to-one correspondence between STs and serovars, whereas larger-scale studies (*n* = 6,887) demonstrated that for a small proportion of STs (four percent of isolates) STs and serotypes were not concordant (Ashton et al., 2016).

MGEs, which include plasmids and bacteriophages, are segments of DNA that encode enzymes and other proteins that mediate the movement of DNA within genomes (Frost et al., 2005). Although the isolates exhibited different backgrounds, including sources, import origins, and sampling time points, they shared 3,625 genes overall. The number of core genes is higher than previous studies on *Salmonella* (Jacobsen et al., 2011; Nova et al., 2019). Some of the genes with unknown functions may be associated with phage infection (Dutilh et al., 2014). The high frequency of *Salmon 118970 sal3* found in the *Salmonella* genome revealed an emerging phage infection in the strains obtained from retail meat. *Salmon 118970 sal3*, with a 39,464 bp genome, was first isolated from water buffalo feces in southern Italy and exhibits lytic activity against *S.* Typhimurium (Paradiso et al., 2016). *Salmon 118970 sal3* has a relatively broad host range, which can be also found in *E. coli* (Li et al., 2020) and *Morganella morganii* (Minnullina et al., 2019). A broad host range phage could potentially be the equivalent of a broad spectrum antibiotic (Ross et al., 2016).

In addition to phages, plasmids are primary vehicles for horizontal gene transfer (HGT) in bacteria (De la Cruz and Davies, 2000). The plasmid replicon IncX1 was the second most common replicon among the isolates. A previous study indicated that the most common types of plasmid replicons of *Salmonella* isolated from food animals in the United States were ColE, IncI1, IncF, and IncX (McMillan et al., 2019). The prevalence of plasmid replicons in the Chinese samples was highly consistent with that found in the United States. The IncX1 plasmid observed in *Salmonella* must remain under surveillance, as many papers have reported the presence of various ARGs, such as *tet(X4)* (Chen et al., 2019), *mcr-1* (He et al., 2020), *mcr-3.1* (Sun et al., 2020), *mcr-5.1* (Guo et al., 2019), *mcr-9* (Cha et al., 2020), and beta-lactamase genes (Zhang X.Z. et al., 2018) in this plasmid. It is clear that infection of humans with *Salmonella* containing such genes will present a difficult problem in the clinic.

The number of possible VFs of *Salmonella* is continuing to increase with the ongoing expansion of the knowledge of the molecular mechanism underlying the pathogenicity of *Salmonella*. For instance, effector proteins involved in survival and replication in so-called *Salmonella*-containing vacuoles have been characterized. Other VFs (i.e., virulence plasmids, toxins, fimbriae, and flagella) have been the subject of studies for decades and might therefore be annotated as “classic” VFs (Van Asten and Van Dijk, 2005). Pathogenic *Salmonella* invades non-phagocytic intestinal epithelial cells by delivering a specialized set of effectors though sophisticated machinery comprising the TTSS, which plays a crucial role in the pathogenesis of *Salmonella* (Que et al., 2013). Although the number of *Salmonella* isolates obtained from chicken was greater than the numbers obtained from other meat types, they harbored fewer genes associated with the TTSS, such as *slrP*, *sopD2*, and *sspH2*. The lack of genes belonging to SPI-2 may affect the intracellular replication function in the host (Figueira and Holden, 2012). Ubiquitous fimbrial adherence genes indicate that a strain is experiencing strong selective pressure (Worley et al., 2018). This phenomenon accelerates the severity of *Salmonella* contamination in retail meat. Although the rate of *Salmonella* contamination was not as high in retail meats such as pork, mutton and beef as it was chicken, the isolates from these other meat types may pose a greater threat to human health.

McDermott and colleagues verified that the correlation of phenotypic resistance with known resistance genes is high (99.0%), except for the resistance to aminoglycosides and beta-lactams (McDermott et al., 2016). More than half of the ARGs (58.0%, 51/88) found in the *Salmonella* genomes of the 341 isolates were associated with aminoglycoside and beta-lactam resistance. The complex relationship between phenotype and genotype should be further studied regarding the functions of not only known ARGs but also the potential genes whose function is modulated by ARGs.

Antimicrobials are delivered to animals for a variety of reasons, including disease treatment, prevention, and control and the promotion of growth/feed efficiency. The use of antimicrobials causes more resistant bacteria to emerge in farm animals and retail meats. Consistent with previous studies in China (Rao et al., 2014), *bla*_*CTX*__–__*M*__–__14_, *bla*_*CTX*__–__*M*__–__55_, and *bla*_*CTX*__–__*M*__–__65_ were the most dominant CTX-M types. It should be noted that the relatively high prevalence of *bla*_*CTX*__–__*M*__–__123_, which has been identified as a novel hybrid of the *bla*_*CTX*__–__*M*__–__1_ and *bla*_*CTX*__–__*M*__–__19_ β-lactamases recovered from *E. coli* isolates in China (He et al., 2013). Our findings validated the results indicating the potential spread of the *bla*_*CTX*__–__*M*__–__123_ gene in China, especially in chicken meat samples (Wang W. et al., 2020). Most of PMQR genes could be found in animals from China (Jacoby et al., 2014), except one isolate, YC300, which harbored the *qnrE1* gene (Monte et al., 2019). The *qnrE1* gene was mainly found in South America (Monte et al., 2019). Colistin, as the last resort for the treatment of infections caused by MDR bacteria, has received extensive attention. In particular, transferable colistin resistance is drawing increasing attention in the study of ARGs (Liu et al., 2016). Our results suggest that the spread of colistin resistance genes, such as the *mcr-1.1* and *mcr-9* genes in chicken, needs to be under strict surveillance in retail markets. The two plasmids IncX4 and IncI2 may increase the fitness of the host and become the major plasmid types driving the dissemination of the *mcr-1* gene (Wu et al., 2018). In the present study, we validated the presence of the *mcr-9* gene commonly on large plasmids (Lin et al., 2020). Colistin has been used for decades in veterinary medicine and as a last-resort drug to treat infections in humans (Kempf et al., 2016). Although colistin sulfate premix was prohibited for use as a growth promoter on April 30th, 2017, in China (Wang Y. et al., 2020), retail meat remains an important source of *mcr*-positive bacteria. In the present study, although not all the genes annotated as *aac(6′)-Iaa* showed the same identity with the reference sequence in the database, they may still have ability to provide aminoglycoside resistance under low nutrient conditions (Cooper et al., 2020). The emergence of MDR *S.* Indiana, a dominant *Salmonella* serovar in China, has raised global awareness because MDR *S.* Indiana has also recently emerged at a rapid pace in other countries (Gong et al., 2019). The high prevalence of *S.* Indiana needs to be given more attention in future studies.

Despite some remaining limitations, the comprehensive information provided by WGS will greatly enhance the monitoring of transmission, HGT, VFs and ARGs. The presence of *S. enterica* carrying virulence and resistance determinants in retail meat compromises food security. The diverse virulence genes and ARGs contained in these isolates may increase the risk of this foodborne pathogen, which warrants constant monitoring. Our data suggested that *Salmonella* contamination should be monitored in not only chicken meat but also pork and duck meat.

## Data Availability Statement

The datasets generated for this study can be found in the online repositories. The names of the repository/repositories and accession number(s) can be found below: https://www.ncbi.nlm.nih.gov/, PRJNA675435.

## Author Contributions

YH, BZ, and CX conceived and designed the project. NL, HH, and CX isolated and sequenced the isolates. YF analyzed the data. YL and CX performed the serotyping and antimicrobial susceptibility testing. NL, YF, and YP wrote the initial manuscript. All authors contributed to the article and approved the submitted version.

## Conflict of Interest

The authors declare that the research was conducted in the absence of any commercial or financial relationships that could be construed as a potential conflict of interest.

## Footnotes

1 https://github.com/tseemann/snp-dists

## Supplementary Material

Supplementary Figure 1 SNP distance among the isolates. **(A)** SNP distance of isolates of *S.* Enteritidis from chicken and duck. **(B)** SNP distance of isolates of *S.* Indiana from chicken and duck. **(C)** SNP distance of isolates of *S*. Thompson from chicken and mutton. **(D)** SNP distance of isolates of *S.* Typhimurium from duck and pork. **(E)** SNP distance of isolates from different sources.

Supplementary Figure 2 Prevalence of the top 10 plasmid replicons **(A)** and top 10 prophages **(B)** at different sampling time points.

Supplementary Figure 3 Correlations between the serovars and the plasmid replicons.

Supplementary Figure 4 Correlations between the serovars and the prophages.

Supplementary Figure 5 Virulence genes in the chromosomes and plasmids. Green: virulence genes in chromosomes; brown: virulence genes in plasmids; purple: plasmid replicons.

Supplementary Figure 6 Correlations between the serovars and the VFs.

Supplementary Figure 7 Numbers of ARGs **(A)** and VFs **(B)** in the *Salmonella* genome belonging to different serovars.
