# Supplementary figures and images for: Genomic Characterization of Salmonella enterica Isolates From Retail Meat in Beijing, China
Source: Front Microbiol. 2021 Apr 7;12:636332. doi: 10.3389/fmicb.2021.636332 (PMC8058101; doi:10.3389/fmicb.2021.636332)

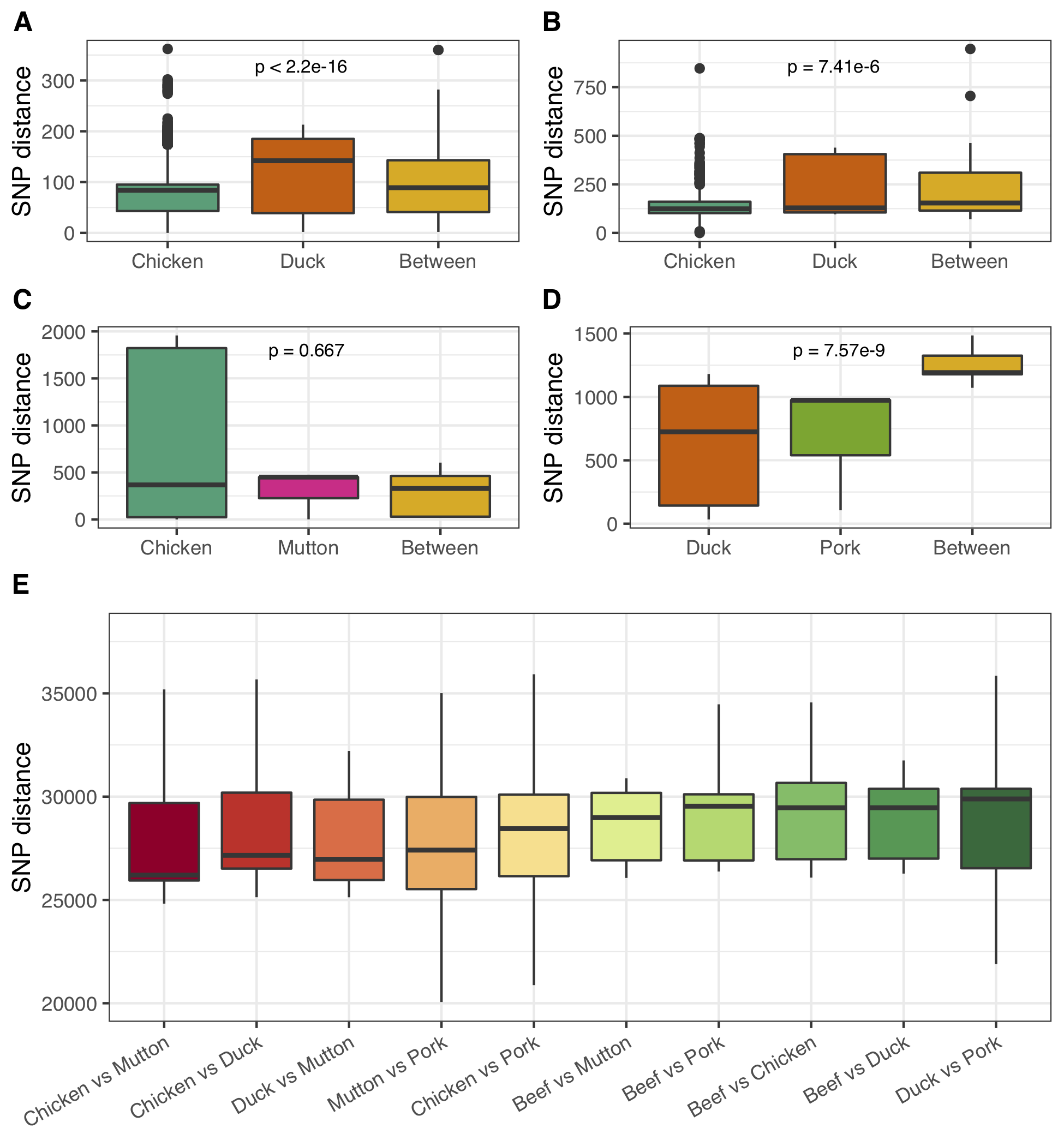

Supplement: Supplementary Figure 1 — SNP distance among the isolates. (A) SNP distance of isolates of S. Enteritidis from chicken and duck. (B) SNP distance of isolates of S. Indiana from chicken and duck. (C) SNP distance of isolates of S. Thompson from chicken and mutton. (D) SNP distance of isolates of S. Typhimurium from duck and pork. (E) SNP distance of isolates from different sources. [file Image_1.TIFF]

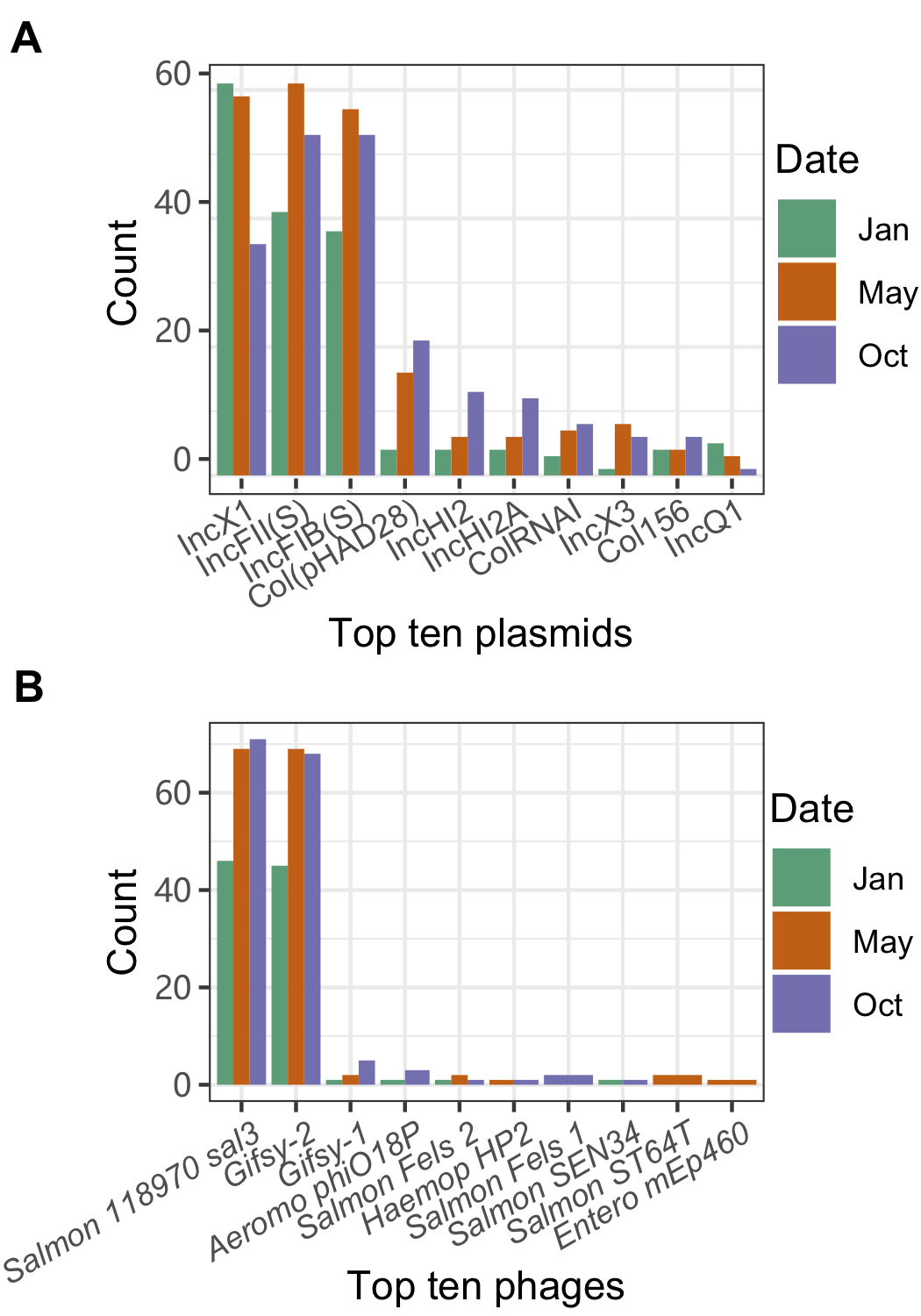

Supplement: Supplementary Figure 2 — Prevalence of the top 10 plasmid replicons (A) and top 10 prophages (B) at different sampling time points. [file Image_2.TIFF]

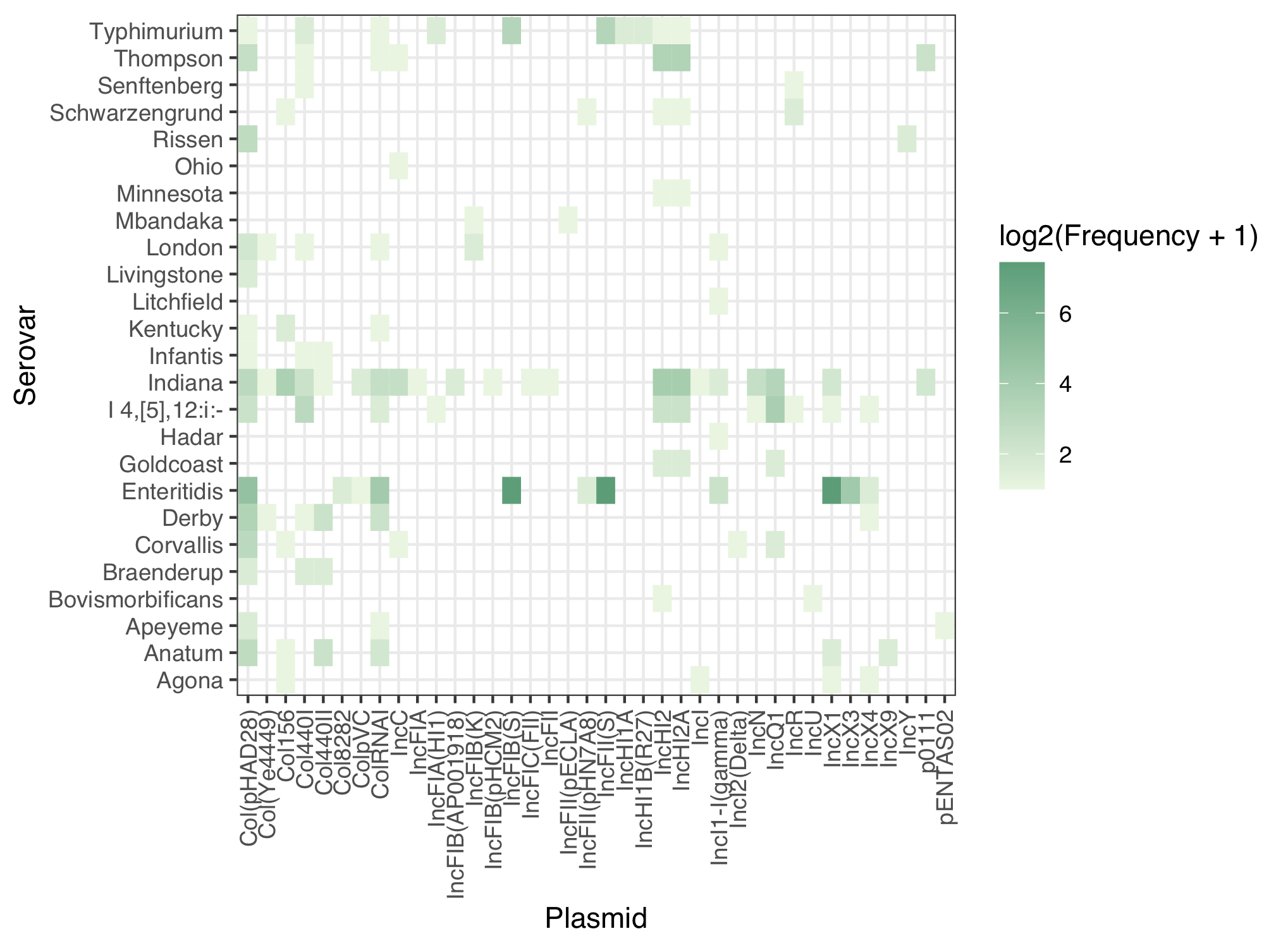

Supplement: Supplementary Figure 3 — Correlations between the serovars and the plasmid replicons. [file Image_3.TIFF]

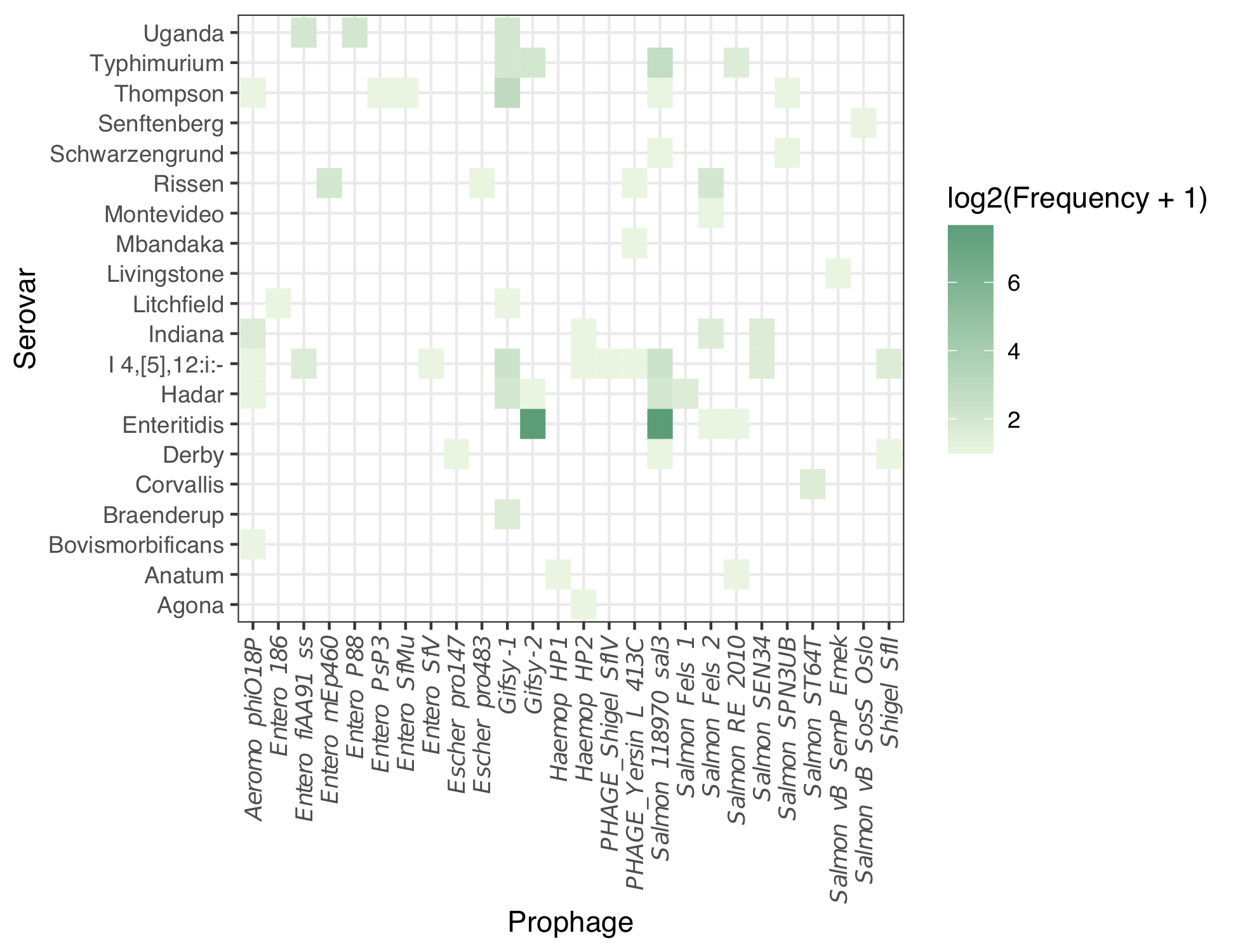

Supplement: Supplementary Figure 4 — Correlations between the serovars and the prophages. [file Image_4.TIFF]

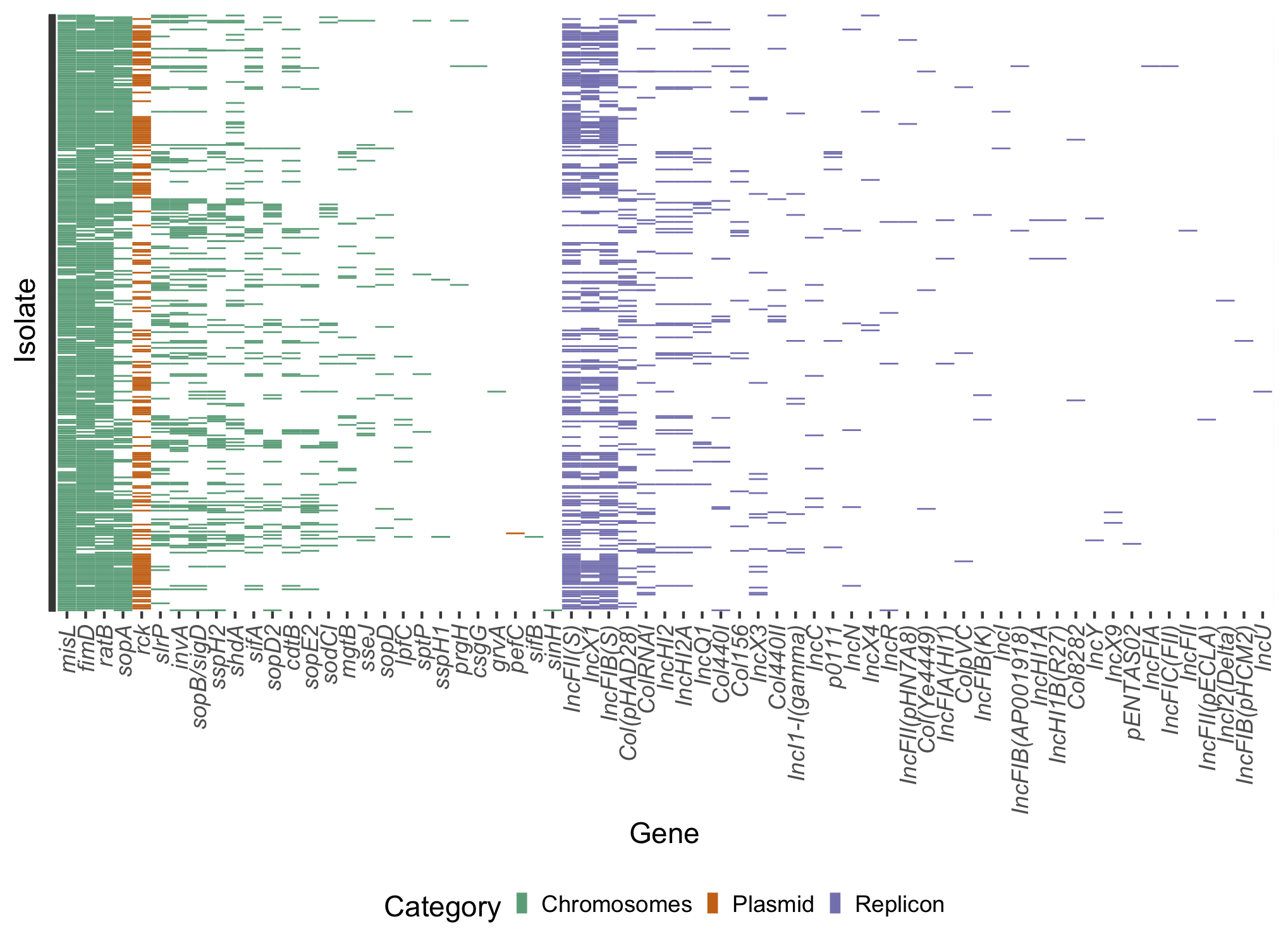

Supplement: Supplementary Figure 5 — Virulence genes in the chromosomes and plasmids. Green: virulence genes in chromosomes; brown: virulence genes in plasmids; purple: plasmid replicons. [file Image_5.TIFF]

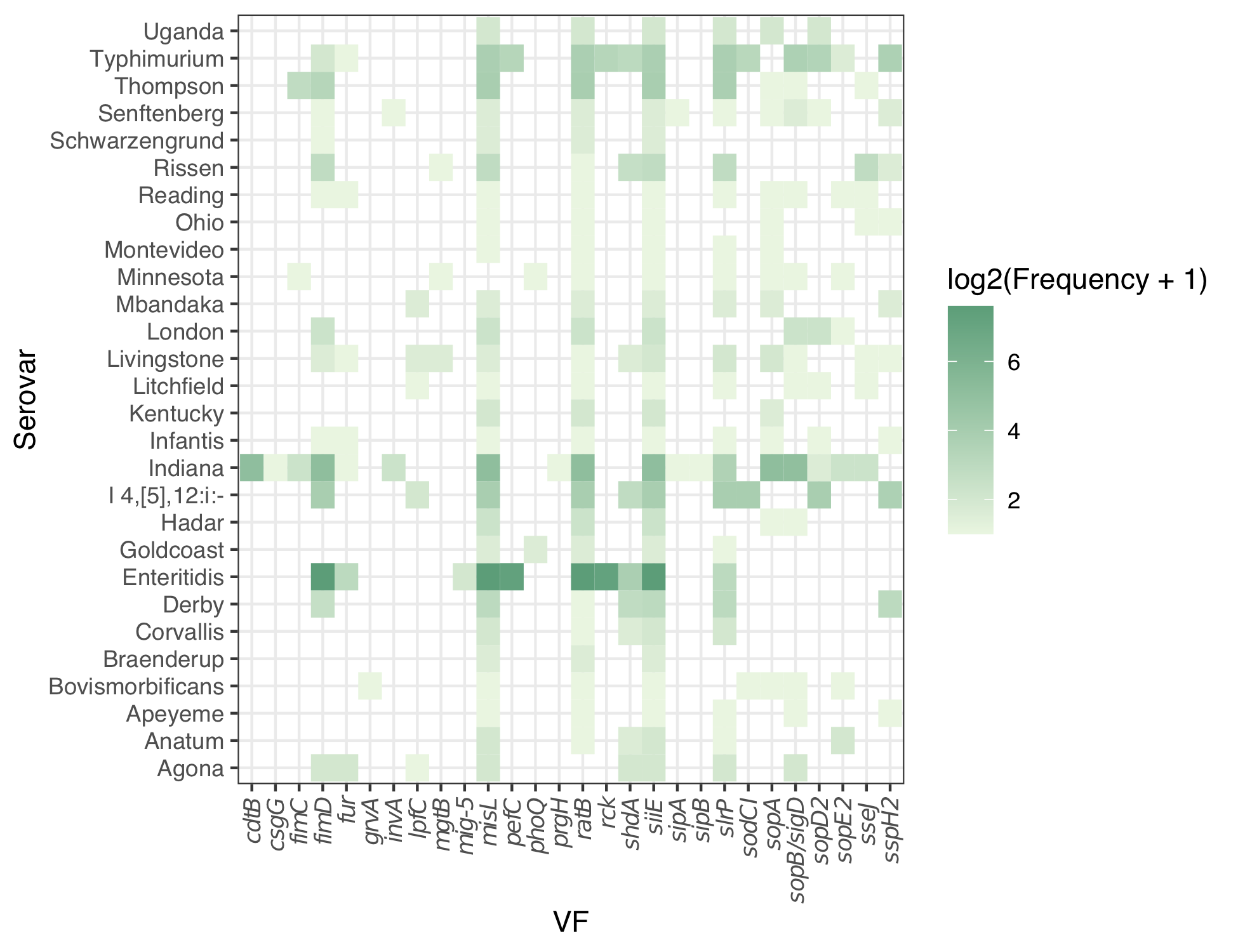

Supplement: Supplementary Figure 6 — Correlations between the serovars and the VFs. [file Image_6.TIFF]

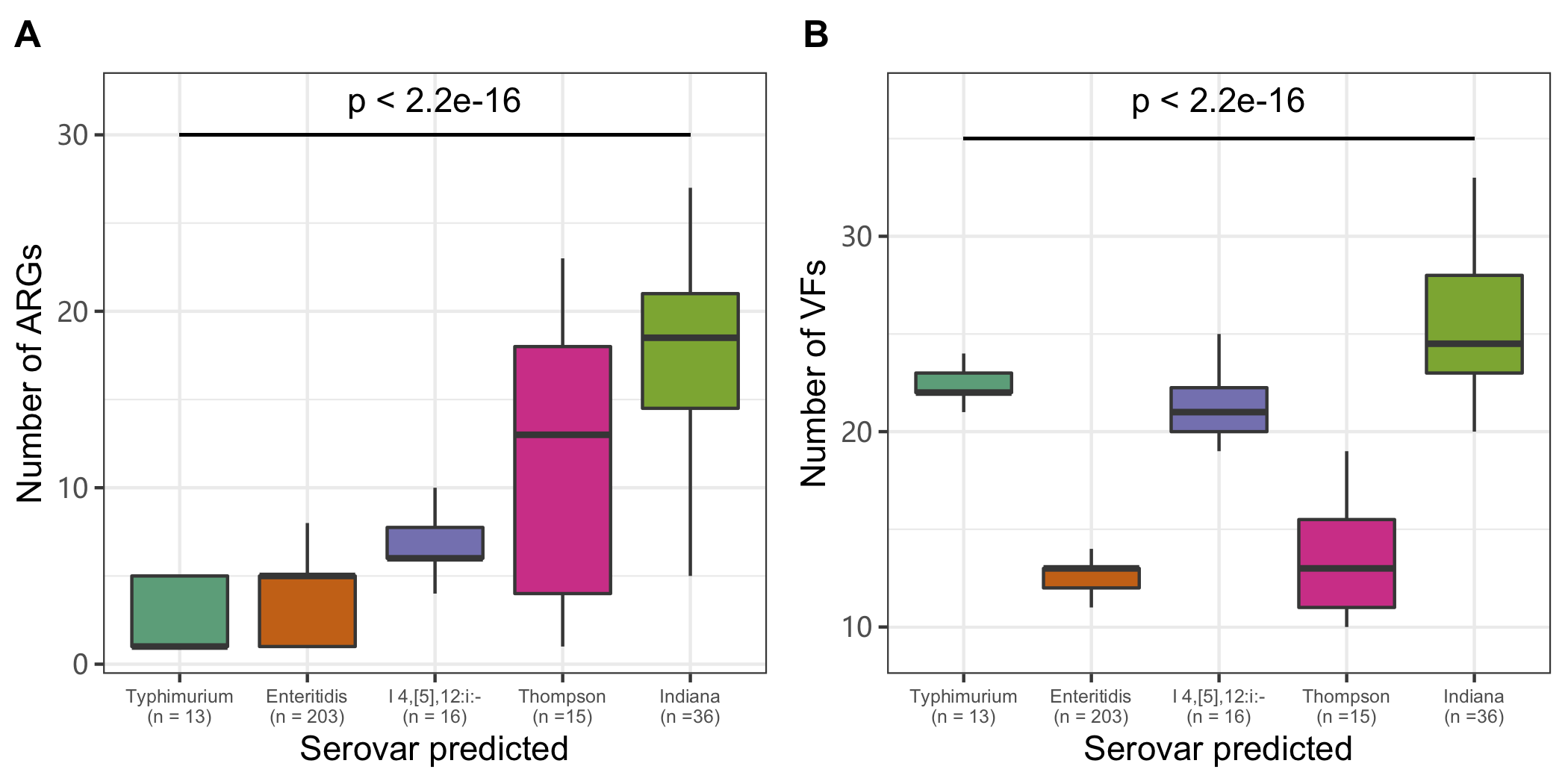

Supplement: Supplementary Figure 7 — Numbers of ARGs (A) and VFs (B) in the Salmonella genome belonging to different serovars. [file Image_7.TIFF]
